# Effects of Comorbidities on Asthma Hospitalization and Mortality Rates: A Systematic Review

**DOI:** 10.1155/2018/6460379

**Published:** 2018-10-01

**Authors:** Masoud Mahdavian, Blake H. Power, Shabnam Asghari, Jordan C. Pike

**Affiliations:** ^1^Division of Respirology, Department of Medicine, Faculty of Medicine, Memorial University of Newfoundland, St. John's, NL, Canada A1C 5B8; ^2^Faculty of Medicine, Memorial University of Newfoundland, St. John's, NL, Canada A1B 3V6; ^3^Librarian II, Eastern Health, Department of Research, St. John's, Canada

## Abstract

**Background:**

Recent studies have shown that patients diagnosed with asthma who have other chronic comorbidities have severely worse medical outcomes. However, the number of available published studies in this field is lacking. The aim of this study was to determine the effects of comorbidities in asthmatic patients based on hospitalization and mortality rates.

**Methods:**

A systematic review was conducted. Data were obtained from the electronic databases PubMed, CINAHL, and Cochrane until June 15, 2018. The primary objective of this study was to determine the effects of comorbidities on asthma hospitalization and mortality. The secondary objective was to analyze the effects of asthma comorbidity with certain chronic diseases, including COPD, obesity, obstructive sleep apnea, mental illness (anxiety and depression), diabetes mellitus, hypertension, myocardial ischemia, rhinitis, and sinusitis on asthma hospitalization and mortality.

**Results:**

From potential 687 articles, only 9 were chosen based on our study inclusion criteria. Almost half of these articles were related to asthma/COPD comorbidity. There were no articles found for hypertension, myocardial ischemia, rhinitis, or sinusitis based on our inclusion/exclusion factors. Each of these 9 published articles had shown an increase in rates of hospitalization, length of stay, and/or mortality, due to asthma-related symptoms, compared to asthma-only patients.

**Conclusion:**

There was determined to be a large discrepancy between the available research for various types of comorbid conditions presenting with asthma that focus on hospitalization and mortality rates. The current available literature suggests a large impact that these comorbid diseases can have on asthma-related symptoms when present together, severely affecting a patient's quality of life. We propose that further research on the effects of these comorbidities on asthma mortality and hospitalization can yield beneficial results to improve the management of asthmatic patients.

## 1. Introduction

Asthma is one of the most common pulmonary diseases among individuals of any age (prevalence of 8% in adults and 15% in children in the United States population) [1]. Recent research has indicated that the presence of a comorbid condition with asthma can have an effect on the patient's quality of life, treatment options, frequency of asthma-related exacerbations, and mortality rates [2]. Although most comorbidities cause some sort of change in patient outcomes, there is little research currently being conducted to analyze these varying effects with asthma, which are crucially important for the patient's quality of life, especially due to differences being present with each varying type of disease comorbidity. The pathological process and outcomes for varying comorbidities between asthma and other severe chronic diseases is still not fully understood, and prevalent links have yet to be established, unlike other comorbid diseases, such as diabetes mellitus with renal disease [2].

This literature review analyzed patients of any age, sex, and ethnicity on their severity of asthma-related symptoms. The main purpose of this study was to determine the effects of comorbidities on mortality and hospitalization rates in patients with asthma. The comorbid diseases included in this study are chronic obstructive pulmonary disease (COPD), obesity, obstructive sleep apnea (OSA), mental illness (depression and anxiety), diabetes mellitus, hypertension, myocardial ischemia, rhinitis, and sinusitis [1]. These chronic diseases were chosen because of their common prevalence in today's population. They have been referred to as some of the most prevalent comorbid diseases with asthma and have been shown to worsen the effect of asthmatic exacerbations in certain populations [1, 3–6]. Each study group is compared with asthma-only patients as a control group. Since randomized control trials are systematically lacking for certain asthma-related comorbidities, we aimed to create a review of the current available literature focusing primarily on the hospitalization and mortality rate differences in asthmatic patients with comorbidities, compared to asthma-only patients.

## 2. Methods

This is a systematic review of peer-reviewed published articles until June 15, 2018. The primary objective of this study is to determine if the presence of a comorbid chronic disease will increase hospitalization and mortality rates in asthmatic patients. The secondary objectives describe how the effects of each specific disease (COPD, obesity, obstructive sleep apnea, diabetes mellitus, cardiovascular disease (hypertension and myocardial ischemia), mental disorders, rhinitis, or sinusitis) will individually increase the rate of hospitalizations and/or mortality in asthmatic patients.

A librarian (JP) conducted a systemic and comprehensive search of electronic databases including PubMed, CINAHL, and Cochrane. The key words were searched independently and combined with either the comorbidity term or list of independently named chronic diseases that were being analyzed and listed above. The search criteria had no exclusion factors for race, age, sex, language, or country of origin of the population studied. All literature reviews, letters to the editor, and commentary articles were excluded. Studies were excluded if they did not include an asthma-only patient group as control. Detailed search strategies for some comorbidities can be found in Table 1. This search strategy was reviewed and agreed upon all authors and was performed for all databases.

**Figure 1:**
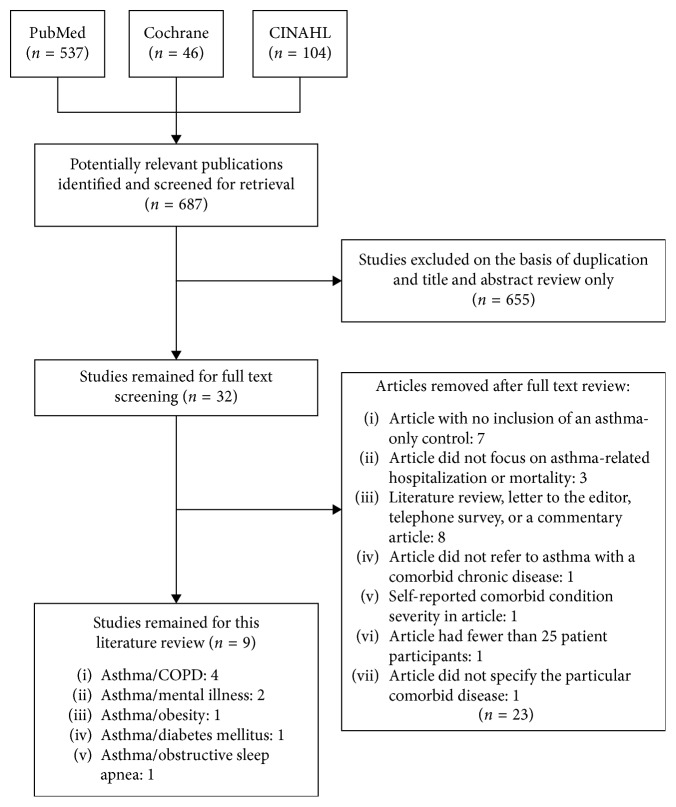
Study flowchart of study selection process for use in literature systematic review.

Two reviewers (BP and MM) screened the articles independently based on title and abstract initially, as stated in Figure 1. Relevant articles were chosen based on these factors and further reviewed in full text. The inclusion factors for full text review were: (1) studies were written fully in English; (2) cohort patients had a chronic disease comorbidity with asthma; (3) the study included a control group of asthma-only patients; (4) the comorbid diseases included at least one of the main chronic diseases chosen to be analyzed for this review, as listed above; (5) the study reported comparison rates of hospitalization and/or mortality due to asthma-related symptoms in both control and cohort patients; and (6) all diseases and symptoms were physician diagnosed and reported from hospital personal, not using an individual symptom survey. Any discrepancies were discussed and resolved between the two reviewers.

This systematic review followed the PRISMA guidelines, and the quality of the studies was evaluated using NIH guidelines and scoring [7].

Manual data were extracted using an excel sheet. Data were extracted independently and entered into the predetermined spreadsheet by BP; then data were compared to ensure correct values. The data extracted from all studies include the name of the first author, year of publication, study design, number of control participants (asthma-only) and number of participants with the comorbid disease, type of comorbid disease studied, and primary outcomes based on hospitalization and/or mortality (Table 2).

## 3. Results

The database search results of the key words used in PubMed are outlined in Table 1, with similar methods used in both CINAHL and Cochrane and for the other comorbid diseases. The screening of the 687 articles obtained from all three databases was done primarily on title and abstract. From this, 32 articles remained for full review and were analyzed based on the particular inclusion factors listed in the methodology section. Of these 32, only 9 articles [8–16] were eventually used for relevance in this systematic review (Figure 1). The characteristics of each of these articles are described in detail in Table 2.

The publication years of the chosen articles ranged from 2010 to 2017. Of these studies, 4 were conducted in the North America (USA and Canada); 2 were conducted in Japan; and 1 was conducted in each Denmark, Australia, and Taiwan. Each study had a varying number of patients involved; however, all had included a particular chronic disease that was present as a comorbidity with previously physician-diagnosed asthma and compared them to asthma-only patients as a control. Table 2 further describes the characteristics of these articles, in terms of the types of study, cohort and control population, and the significant finding based on differences of hospitalizations, length of stay, and/or mortality rates for the cohort patients compared to control.

Of these 9 articles, 3 (33.3%) had reported a significant difference of worsening mortality rates for their asthma comorbidity population compared to the control population. Similarly, 4 articles (44.4%) had stated a significant difference between the number of emergency room visits/hospitalizations, with 1 article (11.1%) indicating increased length of stay (LOS), due to asthma-related symptoms in their cohort population compared to control. 1 article had also stated a significant difference noted in both mortality and hospitalizations rates in their comorbid population compared to control population.

## 4. Discussion

From our knowledge and research, there is currently a lack of available population-based studies for varying asthma comorbidities, even for some of the most common comorbid chronic conditions such as hypertension, in which 31.1% of the world's adult population was diagnosed within 2010 [17]. The pathophysiology of each type of particular asthma comorbidity is different and should be considered as separate when comparing varying chronic conditions. This was also done when discussing the results of the articles analyzed for this review, as follows.

### 4.1. COPD-Asthma

Chronic obstructive pulmonary disorder (COPD) is a well-defined chronic illness for comorbidity with asthma, which is recently referred to as asthma-COPD overlap (ACO) syndrome [8, 9]. Although separately, asthma and COPD are the two most common obstructive airway diseases among adults, there is little research and evidence directly relating to asthma-COPD overlap, even such that it may not even be appropriate to define the overlap as a syndrome and is not common practice for all physicians [9]. Even though it still may not be well defined, ACO has more relevant published articles in terms of mortality rates when compared to other comorbidities of asthma. Table 2 indicates the mortality rate comparisons found in the varying articles. It is unclear, however, the exact range of patients diagnosed with asthma that also have COPD, but previous research mentions the approximate percentage to be between 15 and 25% [8–10]. Patients suffering from ACO were also noted to be significantly older in age (65 years or older), compared to the asthma-only groups in several studies [8, 11]. In terms of hospitalization rates, multiple studies have suggested that patients with ACO had a lower overall quality of life and more frequent hospitalizations due to exacerbations compared to patients with asthma or COPD alone [8, 10]. The economic interest of the information regarding ACO is also important due to the apparent increased frequency of exacerbations and subsequent hospitalizations [18]. Yamauchi et al. analyzed both the length of hospitalization and number of ICU admissions in their large population three-year retrospective study. It was determined that ACO patients (*n*=6279) had a longer length of hospitalization (average of 11 days) over the course of the study when compared to asthma-only patients (*n*=19,865), with an average stay of 9 days [10]. As well, ACO and asthma patients had an ICU admissions rate of 1.8% and 2.0%, respectively, over the study period. These results of similar ICU admissions, in comparison to the length of stay, yield relatively conflicting values compared to the preconceived notion that ACO results in worse exacerbations and more frequent hospitalizations, giving further evidence for the need of more research done in this field (Table 2).

It may be assumed that most comorbidities would increase the rate of mortality compared to each disease on its own because of the combination of several pathological processes at once [8]. Yamauchi et al. showed that over the time interval of July 2010–May 2013 on their national database in Japan, the mortality percentages were 2.3% and 1.2% for the ACO and asthma patients, respectively; however, they interestingly found that mortality rate in COPD (9.7%) is higher than ACO or asthma alone. This relatively large difference in mortality may give light into the relationship between the comorbidity of asthma-COPD, or may, however, also be due to the fact that the general population age of patients with COPD alone is greater than that of asthma and ACO, putting them at a greater risk for mortality throughout the population study [10]. This difference was analyzed further by Baarnes et al., where they reported to have measured mortality rates between asthma, ACO, and COPD patients based also on their age. The total mortality rate, including all individuals of any age for each of the three groups, was stated to be highest amongst patients with ACO (25.9 per 1,000 person-years) compared to COPD and asthma alone (with 23.1 and 7.9 per 1,000 person-years, respectively) (Table 2) [9]. Along with this mortality rate, the actual cause of death that was occurring in patience with ACO was analyzed by Harada et al., which stated how malignant diseases (including varying types of cancer) had a greater cause of mortality than benign diseases, with lung cancer being the greatest for asthma-only patients (*n*=474) and gastrointestinal cancer being greatest for ACO patients (*n*=176) [14].

### 4.2. Asthma-Mental Illness

Multiple articles state the relationship between asthma and anxiety/depression, especially in younger populations, with the common consensus being that the prevalence of anxiety or major depressive disorder (MDD) would increase the likelihood of asthmatic exacerbations and hospital visits [19]. Mental illness is also a common comorbidity with many diseases and unfortunately is quite commonly gone unrecognized and inadequately treated. Very little data were analyzed in terms of the mortality rates in comparison with individuals with a mental illness-asthma comorbidity, but there is research identifying the effects of this comorbidity on asthma-related emergency room (ER) visits, which is related to the increase in asthmatic exacerbations. Research produced by Ahmedani et al. analyzed this relationship of ER visit frequency and showed that those with depression (*n*=187, 32.9% of study population) were at a greater risk of visiting the ER due to asthma-related complications. This study analyzed a population of 568 individuals with asthma and tested for depressive-like symptoms at both the beginning and end of the study time period (1 year). Asthmatic patients that stated they felt symptoms of depression at both time intervals had a greater risk ratio (RR = 1.96) of ER visits compared to those that had stated they felt depressed at the beginning of the study (RR = 0.68) but had resolved by the end of the study period [14].

Anxiety is another form of mental illness that is commonly analyzed with asthma comorbidity, especially in pediatric patients. The common overlap between these two diseases has shown to increase hyperventilation in patients, creating a difficult clinical setting in determining if one disease is the underlying cause or if the comorbidity is the reason for the increase in symptoms [20]. The study by Vuillermin et al. utilized the Spence Children's Anxiety Scale (SCAS) written questionnaire, consisting of 44 questions each scored from 0–3 (with higher scores correlating to more severe anxiety symptoms), to analyze the possible correlation between asthmatic symptoms in children with anxiety. Their results concluded that school-aged children with asthma were at a much higher risk of attaining anxiety symptoms than their nonasthmatic counterparts. This was based on a SCAS-child score difference of 4.8 (95% CI: 2.4 to 7.2) between asthmatic and nonasthmatic children. As well, there was an increased likelihood of children with asthma to have a SCAS-child score within the clinical range. Within the preceding year of the study, the differences between the SCAS-child scores in asthma and nonasthmatic patients were analyzed in terms of hospitalization rates (OR = 5.0; 95% CI: 1.3–8.0) and ER visits (OR = 5.0; 95% CI: 2.0–7.5) [13]. This difference highlights the possible impacts that the asthma-anxiety comorbidity can have on patient symptoms, especially in youth under the age of 18. These studies were beneficial in creating a relationship between asthma-mental illness comorbidity in terms of symptom severity, frequency of ER visits, and hospitalizations. However, further research would be beneficial to understand the affiliation between these two types of diseases.

### 4.3. Asthma-Obesity and Asthma-Obstructive Sleep Apnea

Obesity has become an increasingly substantial health-related problem for individuals, and the possibility for the comorbidity between asthma and obesity (based on the BMI index) worsening asthmatic outcomes, including exacerbation frequency and overall mortality, is currently an extremely important piece of information for patient long-term outcomes. The current literature has a relatively consistent trend towards obesity being a cause of worsening asthmatic symptoms (for both adults and children under the age of 18 years), such as increased exacerbations and asthma-related visits to the ER when both are present together [14, 21]. Grammer et al. analyzed 352 adult subjects in a 12-month, cross-sectional study, with asthma and compared the effects of BMI to patient's answers to an Asthma Quality of Life Questionnaire (AQLQ) (Table 2). The effects of BMI were compared through linear regression, giving a value of *r*=−0.174 for the comparison of AQLQ scores with increased body mass index [21]. Obese patients were also more likely to have asthma-related ER visits throughout the study period compared to nonobese patients (odds ratio = 1.8) [21]. These results were consistent with a study done by Wiesenthal et al., in which 472 children ages 3–10 were analyzed for differences in ER visits between the obese (49%) and nonobese (51%) groups [15]. Over the course of one year, there was a significant difference between the number of asthma-related ER visits in the obese group compared to the nonobese group (1.68 vs. 1.31 visits; *P* value 0.029), and the results showed how obese/overweight children had increased activity limitation and worsening asthmatic symptoms compared to nonobese children [14] (Table 2). These findings continue to add to the apparent relationship between asthma-obesity (at any age) and the frequency of ER visits and overall more difficult quality of life when compared to nonobese asthmatic patients.

Both obesity and obstructive sleep apnea (OSA) have been identified as major comorbid diseases with asthma [15]. As well, OSA has been linked with poor asthma control and an increased burden of disease with both obese and nonobese patients. Becerra et al. conducted a U.S.-based nationwide inpatient study to observe the hospitalization effects of asthmatic patients with OSA, obesity, or a combination of both. Their results indicated a significant increase in the mean hospitalization length of stay (LOS) for both male and female asthmatic patients of any age with both OSA and obesity (4.21 days), followed by a greater LOS in OSA-asthma patients (4.02 days) compared to obesity-asthma patients (3.74 days), with both comorbidities having a greater LOS than asthma-only control patients (3.47 days) (Table 2) [15].

Due to the increasing percent of the population becoming obese, and the apparent underdiagnosis of OSA (primarily in females) [15], more research is required to establish concrete relationships, and guidelines to ensure these patients are receiving the best quality of care.

### 4.4. Asthma-Diabetes

Similar to asthma and obesity, Type 1 diabetes mellitus (T1DM) is a common chronic disorder with early childhood onset and a continuously growing prevalence with health-related issues [22]. However, there still remains little research in the association between T1DM and asthma and the possible worsening affects these chronic diseases may have on one another [16]. Research conducted by Hsiao et al. had found a significantly greater incidence of asthma in patients with T1DM when compared to the general population (6.49 vs. 4.42 per 1,000 person-years for asthma-T1DM prevalence compared to the asthma-only population control, respectively, with HR = 1.34; 95% CI: 1.11–1.62) (Table 2) [16]. This is contradicting to previous studies mentioned by Hsiao et al. stating how T1DM had a negative association between asthmatic symptoms. These conflicting results provide further evidence for the need of more research in this field to allow for a greater understanding and potential options for patient treatments.

### 4.5. Hypertension-Myocardial Ischemia

Throughout our literature search, there were no clinical studies to analyze the effects of hypertension or myocardial ischemia on hospitalization and mortality rates in asthmatic patients based on our inclusion and exclusion criteria. However, a study by Iribarren et al. analyzed a large population of adults for possible correlations of asthma and onsets of other varying types of heart disease. They observed that compared to their nonasthmatic control group, there was a 1.40-fold increase in coronary heart disease (CHD), a 1.20-fold increase in cerebrovascular disease (CVD), a 2.14-fold increase in heart failure, and a 3.28-fold increase in all-cause mortality in patients with an asthma comorbidity [23]. Women were also shown to have greater hazard ratios in all categories compared to men. These findings shed considerable light on the importance of understanding the consequences of having asthma with a comorbid cardiac disease. A study by Tattersall et al. had also reported similar findings, showing that their participants with late-onset asthma (8.7% of their 1269 participants) were associated with an increased risk of CVD events, compared to early-onset or nonasthmatic participants, throughout the decade long study [24]. This research describes the impact that cardiovascular disease can have on asthmatic patients and thus further research should be performed which encompass other prevalent cardiac diseases, such as hypertension and myocardial ischemia.

### 4.6. Rhinitis and Sinusitis

Both allergic rhinitis and sinusitis have been shown to commonly occur as a comorbidity with asthma; however, the understanding of this association has not been fully determined [25]. There were no studies found throughout the literature search that directly analyzed asthmatic-only patients with those that have an asthma-rhinitis or asthma-sinusitis comorbidity. There were other studies, however, that describe the beneficial impacts that the treatment of one of the two diseases could have on the symptoms of both diseases. One such study is by Crystal-Peters et al., who found that those with chronic asthma who were treated nasally for allergic rhinitis (AR), with either inhaled steroids, oral steroids, theophylline, and/or cromolyn, had a significantly lower risk of subsequent pulmonary symptoms, asthma hospitalizations, and ER visits throughout their one-year study [25]. Although the physiology of the association between AR and asthma is still yet to be fully understood, this research allows for further understanding of the economic and patient benefit in treatment of AR, yielding a reduction in health-care utilization for comorbid asthma symptoms.

## 5. Conclusion

Comorbidities are common for many chronic diseases, and the understanding of the increased complexity that arises with these multiple illnesses is crucially important for improved outcomes of patient quality of life. This literature review analyzed the effects of asthma comorbidity with COPD, obesity, obstructive sleep apnea, mental illness (anxiety and depression), diabetes mellitus, hypertension, myocardial ischemia, rhinitis, and sinusitis. From the 687 articles identified initially, only 32 remained after title and abstract exclusion, and of those, 9 remained relevant for this article after full text review, taking into account the specified inclusion and exclusion factors previously stated. Common trends were observed for certain diseases studied, including how patient's ER visits, hospital length of stay, and overall mortality rates increased due to the prevalence of COPD, obesity, OSA, diabetes mellitus, or anxiety/depression. There is a large discrepancy between the amount of available research conducted for the varying comorbidities, with a much higher number of relevant articles pertaining to asthma-COPD than any other comorbid disease. There was overall quite little research regarding certain comorbidities and their effects on asthmatic patient's hospitalization and mortality rates, with some not having any relevant published information (such as hypertension and myocardial ischemia), giving reason for further research to be conducted with both data-based population sets and large randomized trials. These studies will yield a better understanding of asthma and its comorbidities and allow us to provide evidence-based management to reduce hospitalization, mortality rates, and patient suffering.

## Figures and Tables

**Table 1:** Example of the key terms used in PubMed database search for certain comorbid diseases.

Search order	Search term	Number of articles found
#1	Asthma[Mesh] OR asthma^*∗*^[ti]	124,392
#2	Comorbidity[Mesh] OR comorbid^*∗*^[ti]	94,035
#3	Hypertension[Mesh] OR “Diabetes Mellitus”[Mesh] OR “Pulmonary Disease, Chronic Obstructive”[Mesh] OR Anxiety[Mesh] OR “Mental Disorders”[Mesh] OR “Heart Failure”[Mesh] OR “Myocardial Ischemia”[Mesh] OR hypertens^*∗*^[ti] OR high-blood-pressure[ti] OR diabet^*∗*^[ti] OR chronic-obstructive-pulmonary[ti] OR COPD[ti] OR anxiety[ti] OR mental-disorder^*∗*^[ti] OR heart-failure[ti] OR ischemic-heart-disease[ti] OR coronary-heart-disease[ti]	2,206,473
#4	Mortality[Mesh] OR mortality[ti] OR death^*∗*^[ti] OR Hospitalization[Mesh] OR hospitalization^*∗*^[ti] OR hospitalisation^*∗*^[ti] OR length-of-stay^*∗*^[ti] OR LOS[ti]	667,645
#5	Combination of searches: #1 AND (#2 OR #3) AND #4	588
#6	#5 AND English[la]	521

**Table 2:** Study characteristics.

Author (year)	Research type	Population			Study length (years)	C	MD	O	DM	OSA
		Total	Control (asthma-only)	Comorbid condition						
Kendzerska et al. [8]^*∗*^	Retrospective cohort	—	—	—	10	M				
Baarnes et al. [9]	Retrospective cohort	57,053	1,845	662	4	M				
Yamauchi et al. [10]	Retrospective cohort	30,405	19,865	6,279	3	M and ER				
Harada et al. [11]	Retrospective	650	474	176	12	M				
Becerra et al. [12]	Inpatient sample study	179, 789	136,118	8,189	3					LOS
Vuillermin et al. [13]	Cross-sectional analysis	615	410	205	1		ER			
Ahmedani et al. [14]	Prospective follow-up study	568	N/A	187	1		ER			
Wiesenthal et al. [15]	Prospective follow-up study	460	227	233	3			ER		
Hsiao et al. [16]	Retrospective cohort	17,725	766	3,545	13				ER	

Description of results from each selected article regarding the significant differences in hospital/emergency room (ER) visits, mortality (M), and hospital length of stay (LOS) in patient due to the chronic disease comorbidity compared to the asthma-only control. C,  COPD/asthma comorbidity; MD,  mental disorder/asthma comorbidity; O,  obesity/asthma comorbidity; D ,  diabetes mellitus/asthma comorbidity; OSA ,  obstructive sleep apnea. ^*∗*^This paper utilized a population health administrative data for all individuals living in Ontario, Canada (population of approximately 13 million in 2010) that are over the age of 35. ^*∗∗*^No articles were found that discussed the clinical impacts of either hypertension-asthma, myocardial ischemia-asthma, or rhinitis/sinusitis-asthma comorbidity.

## Abbreviations

ACO: Asthma COPD overlap
AR: Allergic rhinitis
AQLQ: Asthma Quality of Life Questionnaire
BMI: Body mass index
CHD: Coronary heart disease
COPD: Chronic obstructive pulmonary disease
CVD: Cardiovascular diseases
ER: Emergency room
ICU: Intensive care unit
LOS: Length of stay
MDD: Major depressive disorder
OSA: Obstructive sleep apnea
SCAS: Spence Children's Anxiety Scale
T1DM: Type 1 diabetes mellitus 4.

## References

[1] de Groot E. P., Duiverman E. J., Brand P. L. P. (2010). Comorbidities of asthma during childhood: possibly important, yet poorly studied. *European Respiratory Journal*.

[2] Gershon A. S., Wang C., Guan J., To T. (2010). Burden of comorbidity in individuals with asthma. *Thorax*.

[3] Sumino K., O’Brian K., Bartle B., Au D. H., Castro M., Lee T. A. (2014). Coexisting chronic conditions associated with mortality and morbidity in adult patients with asthma. *Journal of Asthma*.

[4] Self T. H., Owens R. E., Mancell J., Nahata M. C. (2016). Asthma as a comorbidity in hospitalized patients: a potential missed opportunity to intervene. *Annals of Pharmacotherapy*.

[5] Putcha N., Hansel N. N. (2014). All-cause mortality in asthma. the importance of age, comorbidity, and socioeconomic status. *Annals of the American Thoracic Society*.

[6] Lisspers K., Janson C., Larsson K. (2018). Comorbidity, disease burden and mortality across age groups in a Swedish primary care asthma population: an epidemiological study (PACEHR). *Respiratory Medicine*.

[7] National Heart, Lung and Blood Institute (May 2018). Quality assessment of controlled intervention studies. https://www.nhlbi.nih.gov/health-topics/study-quality-assessment-tool.

[8] Kendzerska T., Sadatsafavi M., Aaron S. D. (2017). Concurrent physician-diagnosed asthma and chronic obstructive pulmonary disease: a population study of prevalence, incidence and mortality. *PLoS One*.

[9] Baarnes C. B., Anderson Z. J., Tjonneland A., Ulrik C. S. (2017). Incidence and long-term outcome of severe asthma-COPD overlap compared to asthma and COPD alone: a 35-year prospective study of 57,053 middle-aged adults. *International Journal of Chronic Obstructive Pulmonary Disease*.

[10] Yamauchi Y., Yasunaga H., Matsui H. (2015). Comparison of in-hospital mortality in patients with COPD, asthma and asthma-COPD overlap exacerbations. *Respirology*.

[11] Harada T., Yamasaki A., Fukushima T. (2015). Causes of death in patients with asthma and asthma-chronic obstructive pulmonary disease overlap syndrome. *International Journal of Chronic Obstructive Pulmonary Disease*.

[12] Becerra M. B., Becerra B. J., Teodorescu M. (2016). Healthcare burden of obstructive sleep apnea and obesity among asthma hospitalizations: results from the U. S.-based nationwide inpatient sample. *Respiratory Medicine*.

[13] Vuillermin P. J., Brennan S. L., Robertson C. F. (2010). Anxiety is more common in children with asthma. *Archives of Disease in Childhood*.

[14] Ahmedani B. K., Peterson E. L., Wells K. E., Keoki Williams L. (2013). Examining the relationship between depression and asthma exacerbations in a prospective follow-up study. *Psychosomatic Medicine*.

[15] Wiesenthal E. N., Fagnano M., Cook S., Halterman J. S. (2016). Asthma and overweight/obese: double trouble for Urban children. *Journal of Asthma*.

[16] Hsiao Y. T., Chen W. C., Liao W. C. (2015). Type 1 diabetes and increased risk of subsequent asthma, a nationwide population-based cohort study. *Medicine*.

[17] Mills K. T., Bundy J. D., Kelly T. N. (2016). Global disparities of hypertension prevalence and control: a systematic analysis of population-based studies from 90 countries. *Circulation*.

[18] Ding B., Enstone A. (2016). Asthma and chronic obstructive pulmonary disease overlap syndrome (ACOS): structured literature review and physician insights. *Expert Review of Respiratory Medicine*.

[19] Schoepf D., Uppal H., Potluri R., Chandran S., Heun R. (2014). Comorbidity and its relevance on general hospital based mortality in major depressive disorder: a naturalistic 12-year follow-up in general hospital admissions. *Journal of Psychiatric Research*.

[20] Keeley D., Osman L. (2001). Dysfunctional breathing and asthma, it is important to tell the difference. *BMJ*.

[21] Grammer L. C., Welss K. B., Kimmel L. G. (2010). Obesity and asthma morbidity in a community-based adult cohort in a large urban area: the Chicago Initiative to Raise Asthma Health Equity (CHIRAH). *Journal of Asthma*.

[22] d’Annunzio G., Tosca M. A., Pistorio A. (2015). Type 1 diabetes mellitus and asthma: a follow-up study. *Allergologia et Immunopathologia*.

[23] Iribarren C., Tolstykh I. V., Miller M. K., Sobel E., Eisner M. D. (2012). Adult asthma and risk of coronary heart disease, cerebrovascular disease, and heart failure: a prospective study of 2 matched cohorts. *American Journal of Epidemiology*.

[24] Tattersall M. C., Barnet J. H., Korcarz C. E., Hagen E. W., Peppard P. E., Stein J. H. (2016). Late-onset asthma predicts cardiovascular disease events: the wisconsin sleep cohort. *Journal of the American Heart Association*.

[25] Crystal-Peters J., Neslusan C., Crown W. H., Torres A. (2002). Treating allergic rhinitis in patients with comorbid asthma: the risk of asthma-related hospitalizations and emergency department visits. *Journal of Allergy and Clinical Immunology*.

